# Measuring Verbal Psychotherapeutic Techniques—A Systematic Review of Intervention Characteristics and Measures

**DOI:** 10.3389/fpsyg.2015.01705

**Published:** 2015-11-10

**Authors:** Antje Gumz, Barbara Treese, Christopher Marx, Bernhard Strauss, Hanna Wendt

**Affiliations:** ^1^Berlin University of PsychologyBerlin, Germany; ^2^Department of Psychosomatic Medicine and Psychotherapy, University Medical Center Hamburg-EppendorfHamburg, Germany; ^3^Institute of Psychosocial Medicine and Psychotherapy, University Hospital JenaJena, Germany

**Keywords:** verbal intervention, technique, skills, measure, psychometric property, systematic review

## Abstract

Language is one of the most important “tools” of psychotherapists. The working mechanisms of verbal therapeutic techniques, however, are still marginally understood. In part, this is due to the lack of a generally acknowledged typology as well as a gold standard for the assessment of verbal techniques, which limits the possibility of conducting studies focusing this topic. The present study reviews measures used in clinical research which assess directly observable dimensions of verbal interventions in a reliable manner. All measures were evaluated with respect to their theoretical foundation, research goals, assessment modes, and various psychometric properties. A systematic search in databases (PubMed, PsycInfo, PsycArticles, PSYNDEX, Web of Science, Embase) followed by an additional “snowballing” search covering the years 1940–2013 yielded *n* = 179 publications eligible for review. Within these publications, 34 measures were identified showing great heterogeneity regarding the aspects under study. Only two measures reached the highest psychometric standards and can be recommended for clinical use without any reservation. Central problems include deficiencies in the systematization of techniques as well as their partly ambiguous and inconsistent definitions. To promote this field of research, it will be important to achieve a consensus concerning the terminology, conceptions and measures of verbal interventions.

## Introduction

The general efficacy of psychotherapy is well established (e.g., Lambert, 2013) based on a variety of reliable and valid measures. Numerous studies demonstrated that psychotherapeutic treatment using different change theories and related to diagnoses is associated with an improvement of symptoms, quality of life and psychological, social and occupational functioning (e.g., Beutler, 2009; Norcross, 2011; Wampold and Imel, 2015). Moreover, there seem to be only minor significant differences of therapeutic effects in different psychotherapy schools (Castonguay and Beutler, 2006; Barber et al., 2013; Wampold and Imel, 2015). However, other findings indicate that there is still a considerable amount of patients who do not benefit from psychotherapy (Lambert, 2013), or even get worse and that the sustainability of therapy effects is limited in certain patient populations (Barlow, 2010).

These findings call for increased research efforts in order to improve mental health care. Accordingly, the focus of psychotherapy research has increasingly shifted from the question “if” psychotherapy is effective to the question what works and how it works (Castonguay, 2013).

### The active ingredients of psychotherapy

What are the active ingredients of psychotherapy? In order to answer this question it is important to decide which variables need to be considered in respective research agendas. Psychotherapy processes can be described with regard to different characteristics (e.g., Orlinsky and Howard, 1987). An important topic in this respect is whether the effect of psychotherapeutic interventions is mainly due to common therapeutic factors or to therapeutic techniques.

Common factors are implicitly relevant in any therapeutic interaction. They are not explicitly anchored in the treatment models of the different schools of psychotherapy, nor are they considered in the treatment models for specific mental disorders (Tschacher et al., 2014). In their Taxonomy Project Tschacher et al. (2014) address the topic of definition und conceptualization of common factors of psychotherapy and delineate different terminological problems and logical inconsistencies. Based on a comprehensive literature search the authors specified all constructs discussed as non-specific or common factors of therapeutic change in psychotherapy research literature by at least two authors. The resulting list comprises 22 common factors (Pfammatter and Tschacher, 2012; Tschacher et al., 2014). These factors include, for example, therapeutic alliance, readiness to change, insight or cognitive restructuring (Connolly Gibbons et al., 2009; Tschacher et al., 2014). In accordance with the Generic Model of Psychotherapy (Orlinsky and Howard, 1987) common factors relate to various aspects of the therapeutic process: Interpersonal (e.g., therapeutic alliance), intrapersonal (e.g., instillation of hope), or clinical aspects (e.g., affective catharsis).

In many cases, techniques which are considered to belong to a particular psychotherapy method, and common factors have been treated as alternatives in explaining therapeutic progress. In other words, it was considered that *either* specific therapeutic interventions *or* common factors can explain the effects of psychotherapy. This dichotomy, however, needs to be questioned. Techniques and common factors cannot be considered independently. Common factors evolve in the context of the therapeutic relationship, which in turn is influenced to a large extent by the techniques. Having this in mind, common factors can be regarded as active principles implemented by the therapist's specific techniques (Castonguay and Beutler, 2006; Pfammatter and Tschacher, 2012; Gumz et al., 2013).

Knowledge about the common factors provides little guidance for the therapist for what can actually be done in the therapy sessions. Empirical knowledge regarding the therapeutic techniques, i.e., regarding the question what therapists mostly do in successful sessions or successful therapies in general, are of great value to psychotherapy, as they allow recommendations of specific practice. Differentiation of therapist behaviors is crucial for specifying the extent to which techniques actually differ and how this ultimately affects treatment outcome (Bergin and Strupp, 2009). Therefore, it is an important desideratum to analyze therapeutic techniques on the level of single sessions or single units of therapeutic interaction to give profound answers to the question of the how of therapeutic effectiveness (Margison et al., 2000; Mergenthaler, 2015).

### What is specifically meant by technique?

To date, there is no general consensus regarding the question what is meant by the term “technique.” Is this what the therapist does or says? Obviously not. The term can also refer to therapeutic attitudes, implicit theories or even patient behavior typical to a therapy method, e.g., warmth and empathy (Bergin and Strupp, 2009), therapeutic abstinence, neutrality, free association technique or regression (Gumz et al., 2014; Tschacher et al., 2014). Some techniques refer to broader descriptions of procedures or settings, e.g., role playing, sculpture work, reflecting team technique and hypnosis. In other cases, “technique” refers to detailed descriptions of single verbal statements such as the verbalization of emotional reactions or transference interpretations (Gumz et al., 2014; Tschacher et al., 2014).

Bergin and Strupp (2009) pointed out that the repertoire of the contemporary psychotherapist includes an impressive list of techniques, which often are employed in combination but more or less intuitively and unsystematically. Schools of therapy differ in their relative emphases upon particular techniques.

In our view, it is possible to draw a distinction between specific and common techniques. The term “specific” means that a certain technique is unique for a particular psychotherapy method. For example, exposure with response prevention is considered as a typical technique of cognitive-behavioral therapists, transference interpretation as a typical technique for psychodynamic therapists and circular questions as a typical technique for systemic therapists. Common techniques are those that are not specific for a respective therapy method. For example, the technique of exploration cannot be related to a specific therapy method. Another example is the verbalization of emotional reactions which is considered as typical technique of humanistic therapists, while psychodynamic therapists use this technique as well (Gumz et al., 2014).

The term “specific” might also mean that a certain technique may be tailored to the treatment of a specific psychiatric disorder. Such a tailored technique is, for example, planning of pleasant activities in the treatment of depressive disorders or keeping a food diary in the treatment of eating disorders (Pfammatter and Tschacher, 2012; Tschacher et al., 2014).

All in all, therapeutic techniques are characterized by numerous very different features, which makes it difficult to establish definite criteria for their assessment (Gumz et al., 2014). One approach to reduce complexity is to focus on single verbal techniques as a first step. On this level, however, an orientation is not easy as well, as there is no generally acknowledged typology of verbal techniques. Types of verbal techniques are described inconsistently with regard to the number and kind of techniques of a certain therapeutic method. Moreover, definitions of categories with the same label partly do not match, or vice versa, identical aspects have differing designations (Brumberg and Gumz, 2012; Gumz et al., 2014).

This can be illustrated using the example of clarification which was analyzed amongst other categories within the framework of a systematic investigation of definitions of psychodynamic intervention techniques in the theoretical literature (Gumz et al., 2014). In most, but not in all of the analyzed sources, clarification was specified as a type of intervention technique of high importance. Different authors consistently specified that the therapeutic aim and a core characteristic of a clarification is to foster the understanding of a phenomenon. This aim is also described as a core characteristic of the concept interpretation. The formal techniques through which this aim is supposed to be achieved, are heterogeneous. Thus, the clarification was described as: (a) The patient is asked to specify and to associate or (b) the patient is asked to describe a phenomenon in more detail or (c) the therapist identifies recurrent topics or themes or (d) the therapist inquires about the patient‘s feelings (e.g., with respect to related associations or parallels) or (e) the therapist describes the effect of the patient's behavior on the therapist or, finally,(f) the therapist rephrases the central ideas of the patient's statement or summarizes these.

In order to clarify what is specifically meant by “therapeutic techniques,” we suggest to systematize on four hierarchical levels:

Level (1) First of all, techniques referring to the therapeutic dialogue need to be distinguished from techniques referring to broader descriptions of procedures or settings or physical exercises, e.g., role playing, sculpture work, reflecting team technique and hypnosis, keeping a food diary in the treatment of eating disorders, or telling the patient to do a breathing exercise.

Level (2) Within the group of techniques referring to the therapeutic dialogue, verbal, and non-verbal techniques can be distinguished. We define verbal techniques as the verbal utterances of the therapist within the therapeutic dialogue. Non-verbal techniques (or behavior, communication) are closely intertwined with verbal information and prosodic features (Madonik, 2001; Pawelczyk, 2011; e.g., mimic signals, affective expressions, movement patterns, see Geißler, 2005).

Level (3) Verbal techniques contain directly observable and latent features. *Directly observable features* relate to the semantic content (“what is said”) of therapeutic utterances or other semantic units (words, sentences, longer segments) which can be rated based on session transcripts or audio recordings. *Latent features* characterize the implicit pragmatic content of the utterances (“what is implicated” or “what is meant”). In order to evaluate latent features of verbal techniques a higher degree of subjective inference is necessary, compared to directly observable features. If a whole session is rated (global coding), the semantic or implicit pragmatic content of all therapeutic utterances is usually aggregated. In consequence, global coding methods generally involve a higher degree of subjective inference because larger amounts of data need to be cognitively aggregated and there are no obligatory rules regarding this aggregation (Heaton et al., 1995). Among the latent features there are, e.g., functional characteristics (e.g., therapist's intentions such as “directing the dialog,” “speaking kindly,” “controlling the affects” or therapeutic attitudes like abstinence, neutrality) or qualitative characteristics (e.g., therapist's empathy, warmth or competence, internal coherence of an intervention).

Level (4) Regarding directly observable features we suggest to differentiate three basic characteristics: 1. *Form* (i.e., the formal and structural manner in which the therapist responds to the patient's experience, behavior or statement, e.g. “closed question,” “interpreting,” “paraphrase”), 2. *Temporal focus* (i.e., the period of time to which the intervention refers, e.g., “childhood,” “present”), and 3. *Thematic content* (i.e., the topic of the intervention, e.g., “therapist,” “mother,” “defenses”).

### Research on verbal therapeutic techniques

Within psychotherapy process research, varying instruments are used to measure therapeutic techniques, each having its own focus, capturing different features, and showing different psychometric characteristics. The findings of empirical studies are highly variable and often inconsistent, making it difficult to draw firm conclusions regarding the nature, processes, and effects of verbal techniques. There is an overwhelming amount of results concerning different techniques from various psychotherapy schools, naturalistic or manualized, gained by means of observational studies or interventional studies and on the basis of very different sample sizes. These studies examined the association of techniques with different factors such as symptom change, therapeutic relationship, therapeutic gains in single sessions or the relationship of specific techniques with therapy- or therapist-related variables. The diversity of these studies is difficult to systematize.

It can be assumed that all these difficulties contributed to the fact that, so far, there is little systematic knowledge regarding the efficacy of therapeutic techniques. Reviews on the subject are either concerned with selected types of techniques (e.g., transference interpretation; Høglend, 2004; Brumberg and Gumz, 2012), methodologically flawed (e.g., Blagys and Hilsenroth, 2000, 2002), or out-dated (Elliott et al., 1987).

An important first step to advance this field of research should be to establish a more solid theoretical as well as methodological framework for the measurement of verbal techniques. As a starting point we suggest to differentiate four basic features of verbal techniques as explained in the previous section: 1. *Form* (observable characteristic), 2. *Temporal focus* (observable characteristic), 3. *Thematic content* (observable characteristic), and 4. *Latent characteristics*. Such a typology can be helpful to review and structure the amount of existing theoretical articles and empirical results regarding categories of verbal therapeutic techniques.

### The current review

Systematic knowledge regarding the question what kind of verbal techniques a therapist should or should not apply in his or her therapeutic work is of major practical importance. Our review attempts an initial clarification of the state of affairs by addressing the question which instruments are available for the assessment of verbal techniques in psychotherapy, which types of techniques are assessed and which psychometric properties the measures have.

We will restrict our review to the directly observable features of verbal techniques (see Section What is Specifically Meant by Technique?). Verbal techniques are the cornerstone of most psychotherapeutic methods. Language is the basic medium through which new information is conveyed, and it is one of the most important “tools” of the therapist (Gumz et al., 2014). Verbal techniques can, in principle, be examined beyond the differences between therapeutic schools. Focusing on verbal techniques, we will concentrate on measures used to assess directly observable dimensions. This means that we will restrict the review on measures which assess the formal, temporal, and thematic dimensions. Measures that exclusively address latent characteristics of therapeutic techniques were not considered in this review, as this would have potentiated the complexity of the research agenda and in the light of the long-term objective to systematically analyze techniques in order to be able to establish the state of the art in this research area. By focusing on explicit features of verbal techniques it is possible to clarify major facets of the research agenda. The next step will then be to extend the focus of research on latent features.

Moreover, an important criterion was that the reviewed measures report reliability.

We hope to contribute to a more consistent understanding of the subject. A more advanced systematization will help researchers to evaluate results as well as to choose existing instruments or to develop new instruments. Moreover, there is a practical benefit of our review: Only if the categories of a measure can be reliably registered by different individuals (interrater reliability), they can later be correctly trained and applied by other clinicians.

Our specific research questions were:

1. What are the characteristics of existing reliable measures assessing the formal, temporal and thematic dimensions of verbal therapeutic techniques?
  - What is the theoretical basis for the measures?
  - How have the measures been developed and which research questions were addressed?
  - What are the scales, categories, or items underlying the measures?
  - Which assessment mode is employed and which rating perspectives are considered?
2. What are the psychometric properties of the measures?

## Methods

### Eligibility criteria

Studies were eligible for inclusion if they met the following criteria:

- The study dealt with verbal therapeutic techniques within a psychotherapeutic setting.
- Verbal therapeutic techniques were assessed at least partially with regard to formal, temporal or thematic features.
- The study was based on ratings of specific psychotherapy sessions.
- The study referred to individual outpatient psychotherapy with adults.
- The study was published in a peer-reviewed journal.
- The study was published in English or German.
- The measure was developed with regard to well-established methods assuring the interrater reliability of the assessment of verbal techniques in order to establish its general applicability, i.e., Intraclass Coefficient Correlations (ICC; Shrout and Fleiss, 1979), (weighted) kappa-values (Cohen, 1960, 1968; Fleiss, 1971; Light, 1971), Finn's r (Finn, 1970, 1972), or Pearson's product-moment correlation coefficient. The procedure of reliability assessment and the reliability values need to be reported in a study published in English or German in a peer-reviewed journal.

### Search procedure and study selection process

A systematic database search (Pubmed, PsycInfo, PsycArticles, PSYINDEX, Web of science, and Embase) for the years from 1940 to 2013 was conducted by one of the authors (B.T.) using the following search terms, their combination and truncation: psychotherapy, psychotherapeutic process, process research, measure, scale, rating, instrument, intervention, therapeutic technique, coding. The resulting records were judged by B.T. regarding titles and abstracts considering the above mentioned eligibility criteria. The 162 resulting studies were judged for eligibility by all authors. In a next step, further relevant articles were identified (by B.T. and H.W.) by screening references of the included articles as well as the excluded reviews (“snowballing,” c.f. Greenhalgh and Peacock, 2005) and by searching identified authors' names and measures (hand search). Additionally, the authors of all measures were contacted to collect coding manuals and further relevant information (non-response to the request was not a criterion for exclusion).

### Psychometric evaluation of measures

**Figure 1:**
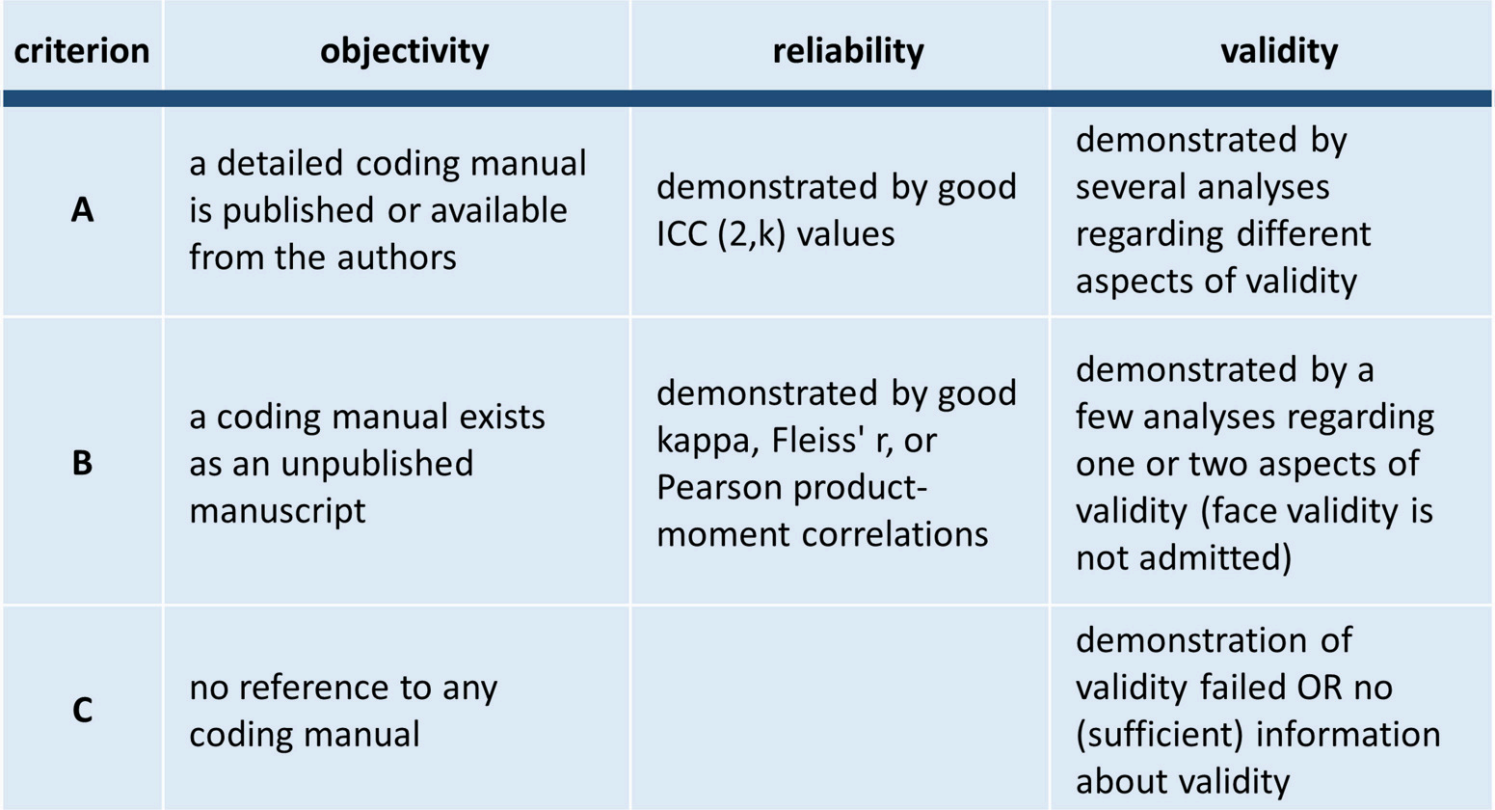
**Criteria for evaluation of the included measures**.

Each measure was evaluated regarding objectivity, reliability, and validity. For each of these criteria, an “A” means that there are no limitations, a “B” indicates some restrictions, and “C” points to severe deficits (see Figure 1 for evaluation criteria). An existing coding manual with clear and unambiguous item/category definitions enables the user to conduct objective ratings. Therefore, we assumed high objectivity when a detailed coding manual was published or available from the authors. Since all included measures report reliability, this aspect is differentiated into two levels with ICC values being the best because they are in line with the highest statistical standards (Bartko and Carpenter, 1975; Tinsley and Weiss, 1975).

## Results

### Study selection

**Figure 2:**
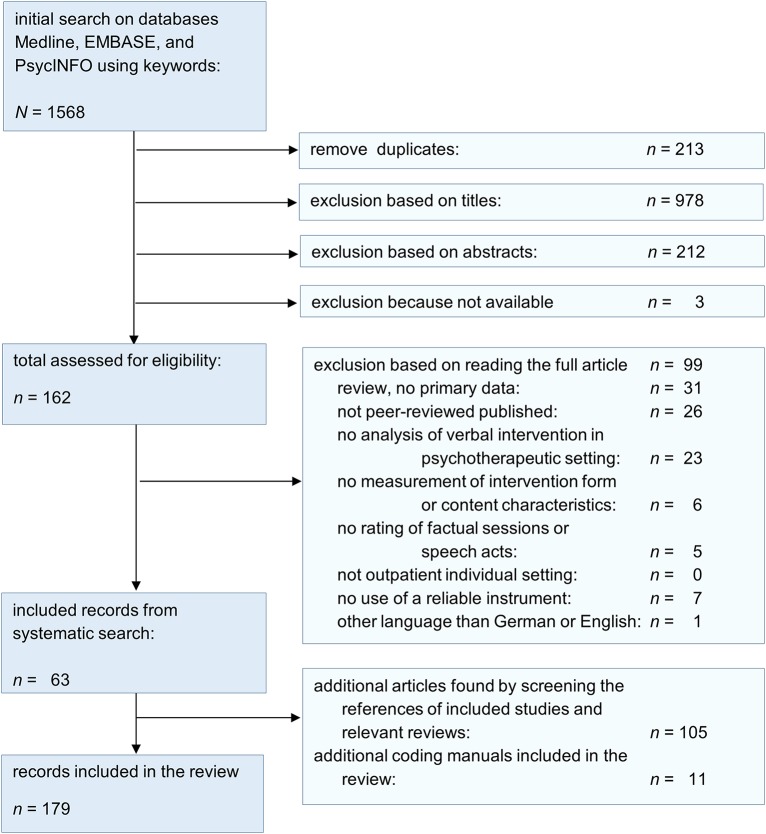
**Flow diagram of study selection process**.

Based on the systematic literature research, 1568 records were found. After screening the titles and abstracts, 162 records were selected for assessment of eligibility. Ninety-nine records had to be excluded: 31 reviews and 26 records were not peer-reviewed (11 book chapters, 15 dissertations), 23 records did not refer to verbal therapeutic techniques, but to other aspects of psychotherapy. Further six records had to be excluded, because the measure did not refer to the intervention form or content characteristics of verbal techniques. Five further records were excluded because they were not used for rating therapy sessions but the general activity of therapists in psychotherapy. Seven measures had to be excluded because of insufficient interrater reliability (e.g., percentages of agreements) or because reliability was not reported at all (Adler and Enelow, 1966; Holzman and Forman, 1966; Karl and Abeles, 1969; Gedo and Schaffer, 1989; Winston et al., 1991; Bucci and Maski, 2007; Hepner et al., 2010). Only one record was published in another language than English or German so that it could not be included. The following snowballing research revealed 116 further relevant records (see Figure 2 for study selection process). In total, 179 articles were included in the present systematic review. They comprise 168 peer-reviewed articles and 11 published coding manuals or scale descriptions and comprise a total of 34 measures. These are presented in Tables 1–**5** in an alphabetical order, separated according to the theoretical orientation and the assessment mode (global measures referring to complete therapy sessions vs. microanalytic measures referring to therapists' single utterances). To maintain a good readability we will refer to the respective measure using its acronym (e.g., YACS for the Yale Adherence and Competence Scale, see Tables 1–**5** for the references of respective acronyms).

**Table 1:** **Global measures referring to psychodynamic therapy**.

**Instrument source**	**Basis Theoretical orientation Objective within the development**	**Short description**	**Assessment mode Raters Definition of scales**	**Valuation**		
				**Objectivity**	**Reliablity**	**Validity**
Adherence/Competence Scale for SE for Cocaine Dependence developed within the NIDA-CCTS (ACS-SEC; Barber et al., 1997)	Luborsky's manual for supportive-expressive treatment (Luborsky, 1984) manual of Mark and Faude (1995); own work (PACS-SE, Barber and Crits-Christoph, 1996) SE To assess the frequency of a technique and the competence with which it is implemented	82 items, 3 subscales: 1. supportive (13 items) 2. expressive (31) 3. cocaine abuse (11) 17 additional items from the original scale PACS-SE	m (7-point Likert scale), session O No coding manual published, description of scales within (Barber et al., 1997)	C	A	B
Columbia Analytic Process Scale (CAPS; Vaughan et al., 1997)	Review of clinical literature, meetings with another study group, TIRS (Bordin, 1966; Piper et al., 1987) AP Specifically designed for use in an outcome study	3 parts: 1. free association (level I, II or III) 2. interpretation vs. non-interpretation (formal intervention, information providing, questions, advice) 3. working through (insight, understanding regarding an observation about the self, fantasy, transference, genetic history)	d, session O Short coding manual within (Vaughan et al., 1997)	C	B	C
Interpretive and Supportive Technique Scale (ISTS; Ogrodniczuk and Piper, 1999)	Interpretive/supportive treatment manuals, literature review, clinical experience PDT Measures interpretive and supportive features of technique for a broad range of dynamically oriented psychotherapies	14 items, 2 scales: 1. supportive techniques: gratify, noninterpretive interventions, guidance, problem solving, explanations, praise, personal information 2. interpretative techniques: pressure, uncomfortable emotions, interpretations, impression of therapist, linking, patient-therapist relationship, impression of others	m (5-point Likert scale), ratings of single interventions complete the overall rating on session-level O Coding manual available from the authors	A	A	B
Therapist Action Scale (TAS) parallel form: Patient Action Scale (PAS) (Hoyt et al., 1981)	Previous therapist-activity rating systems, process-outcome studies, theoretical and clinical background PDT To assess the emphasis of specific actions of therapist and patient	25 items, no subscales e.g., self-concept of patient, expression of feelings, prescription/advise given, future plans of patient	d, m, session O No coding manual published; categories within (Hoyt et al., 1981)	C	B	B
Therapist Verbal Interventions Inventory (TVII; Koenigsberg et al., 1985)	Review of instruments, interventions described in literature, clinical experience PDT To measure the range of interventions used by psychodynamically oriented therapists in therapy of patients with borderline personality disorder	35 items, 20 categories (further specified into 4 subcategories: provides factual information, seeks clarification, confronts, interprets) e.g., gives advice, offers sympathy, focus on external reality	d, 15-min segment O Coding manual exists as an unpublished manuscript	B	B	C
Vanderbilt Therapeutic Strategies Scale (VTSS; Butler et al., 1995)	Manual for time-limited PDT, literature on psychodynamic technique Time-limited PDT Adherence scale to differentiate between experienced and unexperienced therapists	21 items, 2 subscales: 1. interviewing style (IS, 12 items) 2. TDLP-specific strategies (SS, 9 items)	m (5-point Likert scale), session O Coding manual exists as unpublished manuscript	B	A	B

*m, metric; d, dichotomous; O, observer; SE, supportive-expressive dynamic psychotherapy; AP, analytic psychotherapy; PACS-SE, Penn Adherence-Competence Scale for Supportive-Expressive therapy; PDT, psychodynamic therapy; TIRS, Therapist Intervention Rating System; TDLP, Time-limited- dynamic psychotherapy; NIDA-CCTS, National Institute on Drug Abuse-Collaborative Cocaine Treatment Study. Evaluation criteria: A, no limitations; B, some restrictions; C, severe deficits (see text of the manuscript for details)*.

### Theoretical orientation

Eighteen of the included measures refer to a specific theoretical orientation. Most of these are derived from psychodynamic therapy (see Table 1 for global and Table 2 for microanalytic measures). Within these, two measures are specially developed for use in analytic therapy, two for supportive-expressive therapy and one for time-limited psychodynamic therapy, the other seven measures can be generally used in dynamic psychotherapy. Only three measures refer to cognitive-behavioral orientated therapy or to a specific setting (drug counseling, treatment for substance use disorders, motivational interview), respectively (see Table 3). Sixteen measures were classified as “pantheoretical,” because they refer to several different theoretical orientations (see Table 4 for global and Table 5 for microanalytic measures).

**Table 2:** **Microanalytic measures referring to psychodynamic therapy**.

**Instrument source**	**Basis Theoretical orientation Objective within the development**	**Short description**	**Assessment mode Raters Definition of scales**	**Valuation**		
				**Objectivity**	**Reliability**	**Validity**
Analytic process scales (APS; Waldron et al., 2004a)	18 years of bottom-up development, regular meetings over several years; PIRS (Cooper and Bond, 1992; Milbrath et al., 1999), VPPS (O'Malley et al., 1983), TVII (Koenigsberg et al., 1985, 1988), Psychotherapy Process Q-Set (Jones and Windholz, 1990) AP Examine changes occurring over time in psycho-analytic treatments and what features of patient and analyst account for these changes	4 categories: 1. types of intervention (encourage elaboration, clarify, make an interpretation, provide support) 2. aims of intervention (patient's defense, patient's reaction to the analyst/analytic situation, patient's conflicts, romantic/sexual issues, aggressive/hostile issues, developmental focus, self-esteem issues) 3. other characteristics (confronting, feelings of the analyst, analyst's intervention amicable, analyst's intervention hostile) 4. quality of the intervention (follow the patient's immediate emotional focus, good intervention)	m (5-point Likert scale), segment of session O APS Coding Manual (80 pages) available at www.ipa.org.uk or from the author: woodywald@earthlink.net	A	B	B
Coding of therapist statement (Connolly et al., 1998)	/ SE To classify therapist statements into general categories consistent with the techniques of SE therapy	4 categories: 1. interpretation 2. clarification 3. question 4. other; for interpretation, additionally time frame (childhood past, adult past, present) and persons included (therapist, parents, significant other, self, no other person) are detected	d, speaking turn O No coding manual pub-lished; definition of categories in (Connolly et al., 1998)	C	A	B
Malan intervention typology (MIT; Malan, 1963, cited from Silberschatz et al., 1986)	/ PDT To assess interpretations and their impact on therapy outcome	2 scales: 1. interpretation (parent, other, self; transference vs. non-transference) 2. non-interpretation	d, statement O No coding manual published; description within (Malan, 1963, 1976)	C	B	C
Psychotherapy Interaction Coding System (PIC; McCullough, 1988, cited from Town et al., 2012b)	/ PDT To examine therapist interventions and the impact of these interventions in dynamic orientated therapies	2 scales: 1. 8 process codes: question, information, self-disclosure, clarification, directive, support, interpretation, confrontation 2. 4 content codes: defenses, anxieties, impulse/feeling, no content	d, response O Coding manual: (McCullough, 1988); description of categories: (Town et al., 2012a)	A	B	C
Psychodynamic Interventions Rating Scale (PIRS; Cooper and Bond, 1992, cited from Milbrath et al., 1999)	Among others: ITS (Gaston and Ring, 1992) PDT To examine microprocesses in expressive and psychodynamic therapies regarding specific types of interpretive interventions	2 scales: 1. interpretive interventions: defense, transference 2. supportive interventions: acknowledgments, clarification, questions, associations, reflections, work-enhancing strategies, supporting strategies, contractual arrangements	d, m for interpretative interventions (5-point Likert scale), utterance O Coding manual exists as unpub-lished manual; categories within (Banon et al., 2001)	B	A	B
Therapist intervention rating system (TIRS; Piper et al., 1987)	Review of existing rating scales, (e.g., (Strupp, 1966; Luborsky et al., 1979; Marziali and Sullivan, 1980); PDT) To reliably identify interpretations (intervention defined as “interpretive” when there is reference to a “dynamic component”)	10 categories: 1. noninterventions 4 noninterpretive lower categories without reference to patient's experience: 2. formal 3. information providing 4. information requesting 5. directive 1 noninterpretive lower category (with partial reference to patient's experience): 6. nondynamic component 4 interpretive upper categories with reference to patient's internal conflict: 7. single dynamic component 8. double dynamic component 9. triple dynamic component 10. quadruple dynamic component (dynamic components: wish, fear, defensive process, dynamic expression)	d, statement O Coding manual exists as unpublished manuscript, available from the first author; description of categories with examples within (Piper et al., 1987)	A	B	C

*m, metric; d, dichotomous; O, observer; SE, supportive-expressive dynamic psychotherapy; AP, analytic psychotherapy; PDT, psychodynamic therapy; PIRS, Psychodynamic Intervention Rating Scale; VPPS, Vanderbilt Psychotherapy Process Scale; TVII, Therapist verbal Interventions Inventory. Evaluation criteria: A, no limitations; B, some restrictions; C, severe deficits (see text of the manuscript for details)*.

**Table 3:** **Measures referring to cognitive-behavioral orientated therapy or to a specific setting**.

**Instrument source**	**Basis Theoretical orientation Objective within the development**	**Short description**	**Assessment mode Raters Definition of scales**	**Valuation**		
				**Objectivity**	**Reliability**	**Validity**
**BEHAVIORAL ORIENTATED THERAPY—GLOBAL CODING**						
Cognitive Therapy Adherence-Competence Scale developed within the NIDA-CCTS (CTACS; Liese et al., 1995, cited from Barber et al., 2003)	Cognitive Therapy Scale (CTS; Young and Beck, 1980), CSPRS (Hollon et al., unpublished work), Current CT treatment manuals, format based on PACS-SE (Barber and Crits-Christoph, 1996) CT To provide a wider coverage of cognitive therapists' activities	21 items, no subscales e.g., guided discovery, asking for evidence/alternative views, eliciting automatic thoughts, addressing key issues each item is rated separately for AD and COM	m (7-point Likert scale), session O No coding manual published, description of scales within (Barber et al., 2003)	C	A	B
Cognitive Therapy Scale(CTS-R; Young and Beck, 1980, cited from Blackburn et al., 2001 diverse revisions, among others by Blackburn et al., 2001)	cognitive therapy manual (Beck et al., 1979) CT to measure competence in CT (for depression)	11 items, 2 subscales: 1. general skills subscale: agenda setting, eliciting feedback, collaboration, pacing and efficient use of time, emphatic skills, professionalism 2. specific cognitive therapy skills subscale: guided discovery, conceptualization, focus on key cognitions, application of cognitive techniques, application of behavioral techniques, use of homework	m (7-point scale), session O Coding manual available at: http://members.academyofct.org/files/documentlibrary/CTRS%281%29.pdf	A	A	B
**BEHAVIORAL ORIENTATED THERAPY—MICROANALYTIC CODING**						
Coding system for the Interaction in Psychotherapy (CIP; Schindler et al., 1989)	Review of literature; Verbal Response Modes (Stiles, 1978); Response Mode System (Snyder, 1945; Strupp, 1957; Hill et al., 1981; Elliott et al., 1982; Hahlweg et al., 1984) CBT To assess the patient-therapist interaction in behavior therapy	19 items for therapist verbal behavior modes: 6 therapist dimensions: empathy (3 items), support (3 items), exploration (2 items), explanation (4 items), directivity (2 items), classification (3 items), 2 further categories: silence, remainder category	d, sentence O Coding manual existent as an unpublished manual; description of categories within (Schindler et al., 1989)	B	B	C
**SPECIFIC SETTING**						
Adherence/competence scale for IDC for cocaine Dependence developed within the NIDA-CCTS (ACS-IDCCD; Barber et al., 1996)	Individual drug counseling (IDC) manual (Mercer and Woddy, 1992) IDC To assess treatment AD and COM in individual drug counseling (IDC) for the treatment of cocaine-dependence	43 items, 5 subscales: 1. monitoring drug use behavior (6 items), 2. encouraging abstinence (8 items), 3. encouraging 12-step participation (5 items) 4. relapse prevention (5 items) 5. educating the client (5 items) 1 additional section: psychodynamic/cognitive interventions (4 items) 10 additional items without categorization each item is rated separately for AD and COM	m (7-point Likert scale), session O Coding manual available from the author	A	A	B
Sequential Code for Observing Process Change (SCOPE; Moyers and Martin, 2006)	Motivational Interviewing Skill Code (MISC, Moyers et al., 2003; Miller et al., 2008), Commitment Language Coding System (Amrhein et al., 2003) MI to evaluate the quality of motivational interviewing, to measure transition Probabilities and to categorize MI-relevant Verbal behavior between therapist behavior and client behavior during MI sessions	46 items (30 therapist, 16 client items), 8 categories: client categories: 1. neutral utterances, 2. positive commitment language (change-talk), 3. negative commitment language (sustain-talk) counselors categories: 1. questions (open vs. closed) 2. reflections (simple vs. complex), 3. MI-consistent (affirmations, emphasizing the control of the client, seeking permission prior to providing information or advice, offering support), 4. MI-inconsistent (giving advice without permission, confronting clients with information about the target behavior, directing certain courses of action, stating an opinion about client behavior or attitudes, warning clients about consequences of the target behavior) 5. other	d, utterance O MISC coding manual available at: casaa.unm.edu	A	B	B
Yale Adherence and Competence Scale II (YACS; Carroll et al., 2000)	Review of session videotapes and treatment manuals To rate therapist AD and COM in delivering behavioral treatments for substance use disorders	40 items, 6 subscales: 1. assessment (5 items), 2. general support (5 items), 3. goals of treatment (5 items), 4. clinical management (CM, 10 items), 5. twelve step facilitation (TSF, 9 items), 6. cognitive-behavioral treatment (CBT, 6 items) each item is rated separately for AD and COM	m (5-point Likert scale), session O Coding manual available at: http://www.mirecc.va.gov/visn1/docs/products/Yale_Adherence_and_Competence_Scale_II_Guidelines.pdf	A	A	A

*m, metric; d, dichotomous; O, observer; CT, cognitive therapy, CM, clinical management; AD, adherence, COM, competence; CBT, cognitive behavioral therapy; CM, clinical management; TSF, twelve step facilitation; IDC, individual drug counseling; SD, standard deviation; SE, supportive expressive therapy; NIDA-CCTS, National Institute on Drug Abuse–Collaborative Cocaine Treatment Study; CSPRS, Collaboratory Study Psychotherapy Rating Scale; PACS-SE, Penn Adherence-Competence Scale for Supportive-Expressive Therapy; MI, motivational interviewing. Evaluation criteria: A, no limitations; B, some restrictions; C, severe deficits (see text of the manuscript for details)*.

**Table 4:** **Pantheoretical global measures**.

**Instrument source**	**Basis Theoretical orientation Objective within the development**	**Short description**	**Assessment mode Raters Definition of scales**	**Valuation**		
				**Objectivity**	**Reliability**	**Validity**
Comparative Psychotherapy Process Scale (CPPS; Hilsenroth et al., 2005)	Two reviews of the empirical comparative psychotherapy process literature (Blagys and Hilsenroth, 2000, 2002) PI, CBT To classify and compare PI and CBT treatments and to assess therapist activity, process and techniques used in a therapy session	20 items, 2 subscales: 1. psychodynamic-interpersonal subscale (10 items), 2. cognitive-behavioral subscale (10 items)	m (7-point Likert scale), session P,T,O Coding manual available at http://supp.apa.org/psycarticles/supplemental/pst_42_3_340/pst_42_3_340_supp.html	A	A	A
Collaborative Study Psychotherapy Rating Scale (Form 6) (CSPRS; Hollon et al., 1984, cited from Hill et al., 1992)	Consultation of trainers of treatment modalities, several treatment manuals, based on MTRS (DeRubeis et al., 1982) CBT, IPT, CM To distinguish sessions of cognitive therapy, interpersonal psychotherapy, and clinical management pharmacotherapy	96 items, 8 scales: 3 modality-specific scales: 1. CBT scale (28 items, 6 subscales), 2. IPT scale (28, items, 7 subscales), 3. CM scale (20 items, 5 subscales) 3 tangential modality scales (8 items, 2 CBT, 1 IPT subscale) 2 non-modality-specific scales (12 items, 2 subscales: facilitative condition (FC, 8 items), explicit directiveness (ED, 4 items)	m (7-point Likert scale), session O Coding manual (Evans et al., 1984)	A	A	B
Sheffield Psychotherapy Rating Scale (SPRS; Shapiro and Startup, 1990, cited from Startup and Shapiro, 1993)	41 items of CSPRS, (3 items slightly changed), 16 new items ET, CBT, IPT Designed to rate adherence of therapists to exploratory therapy (psychodynamic/experiential therapy with an interpersonal focus)	59 Items, 3 scales: 1. exploratory therapy scale (ET, 19 items (4 items from the IPT scale of CSPRS, 14 new items) 6 subscales) 2. prescriptive scale (P, 32 items; CBT + 2 of tangential modality scale of CSPRS + 2 new items) 3. FC scale of CSPRS (8 items)	m (7-point Likert scale), session O Raters' manual (Shapiro and Startup, 1990), description of items within (Startup and Shapiro, 1993)	A	A	B
Multitheoretical List of Therapeutic Interventions (MULTI; McCarthy and Barber, 2009)	Treatment manuals, therapy books, adherence measures, theoretical and review articles, experts To assess therapeutic interventions from different psychotherapy orientations and from the perspective of patients, therapists, and observers	60 items, 8 subscales: 1. behavioral (BT, 15 items), 2. cognitive (CT, 16 items), 3. dialectical-behavioral (DBT, 8 items), 4. interpersonal (IPT, 7 items), 5. person centered (PC, 7 items), 6. psychodynamic (PD, 12 items), 7. process-experiential (PE, 9 items), 8. common factors (CF, 7 items) (several items are unspecific and belong to multiple subscales)	m (5-point rating scale), session P,T,O No coding manual published	C	A	B
Psychotherapy process Q set (PQS; Jones, 1985, cited from Jones et al., 1987)	Search of extant process measures, discussions with research-oriented clinicians, bottom-up development To provide a standard language and rating procedure for classification of the therapy process and to systematically characterrize a wide range of patient-therapist interactions	100 items, no subscales: 3 types of items: 1. patient attitude and behavior or experience (40 items), 2. therapist's actions and attitudes (41 items), 3. nature of the interaction in the dyad or the climate or atmosphere (19 items)	Q method: items are sorted on a continuum from 1 = least to 9 = most characteristic (5 = neutral/irrelevant), session O Coding manual exists as unpub-lished manual (Jones, 1985)	B	B	B
Therapist Interventions and Qualities Inventory – therapist form (TIQI-T) (TIQI-P; patient form Bøgwald, 2001)	Previous research of literature, feedback of experienced clinicians, Barrett-Lennard Relationship Inventory (1962) To assess specific therapist interventions and unspe-cific interpersonal skills and qualities that have been emphasized in other instruments e.g., Barrett-Lennard Relationship Inventory	36 items, no subscales: e.g.,: encouraged the patient to explore uncomfortable emotions, made interpretations, encouraged patient to talk about what others might feel toward patient, questioned patient about his/her feeling toward therapist	m (5-point Likert scale), session T No coding manual published	C	B	C
Vanderbilt Psychotherapy Process Scale Developed within Vanderbilt Psychotherapy Research Project (VPPS; O'Malley et al., 1983; Strauss et al., 1992)	Based on *Therapy Session Report* (Orlinsky and Howard, 1967, 1975) and further development; (Gomes-Schwartz and Schwartz, 1978) To assess relevant aspects within the therapeutic process Three domains: exploratory processes, therapist-offered relationship, and client involvement	64 items, 8 subscales: 1. client participation (8 items), 2. client hostility (6 items), 3. client dependency (6 items), 4. therapist warmth and friendliness (9 items), 5. negative therapist attitude (6 items), 6. client exploration (7 items), 7. therapist exploration (13 items), 8. client psychic distress (9 items)	m, 15-minute segment of session or entire session O Coding manual existing as an unpublished manuscript	B	B	B

*m, metric; P, patient; T, therapist; O, observer; CBT, cognitive-behavioral therapy; PI, psychodynamic-interpersonal therapy; IPT, interpersonal therapy; CM, clinical management; ET, exploratory therapy; FC, Facilitative conditions. Evaluation criteria: A, no limitations; B, some restrictions; C, severe deficits (see text of the manuscript for details)*.

**Table 5:** **Pantheoretical microanalytic measures**.

**Instrument source**	**Basis Theoretical orientation Objective within the development**	**Short description**	**Assessment mode Raters Definition of scales**	**Valuation**		
				**Objectivity**	**Reliability**	**Validity**
Coding System of Therapeutic Focus (CSTF; Goldfried et al., 1989, cited from Kerr et al., 1992)	Consultation of researchers /practitioners from CBT and IPT, preliminary scoring of therapeutic vignettes from other psychotherapy researchers and published transcripts appearing in the literature *CBT, PI* To examine the content of the therapist's in-session focus and to facilitate comparative process analyses across therapeutic orientations	40 items, 6 categories: 1. components of functioning (10 items), 2. general interventions (7 items), 3. intrapersonal links (3 items), 4. interpersonal links (5 items), 5. persons involved (9 items), 6. time frame (6 items)	d, utterance O Original article is an unpublished manuscript; for description of scales in detail, see e.g., (Goldsamt et al., 1992); no coding manual published	C	A	B
Helping Skills System (HSS; Hill and O'Brien, 1999, cited from Hess et al., 2006)	Substantially revised version of the HCVRCS (Hill Counselor Verbal Response Category System, Hill, 1978) To classify counselor's responses without regard to the quality of the message	12 categories; 3 subscales: 1. exploration skills (approval/reassurance, closed question, open question, restatement, reflection of feelings), 2. insight skills (challenge, interpretation, self-disclosure, immediacy), 3. action skills (information, direct guidance). residual category	d, grammatical sentence O Coding manual available at http://forms.apa.org/books/supp/hill3/pdf/student/webformE.pdf	A	B	B
Hill Counse-lor Verbal Response Category System–Revised (HCVRCS–R; Friedlander, 1982)	Revised version of HCVRCS (Hill, 1978) Conceptually and methodologically More rigorous than the original version	9 categories: encouragement/approval/reassurance, reflection/restatement, self-disclosure, confrontation, interpretation, providing information, information seeking, direct guidance/ advice, unclassifiable	d, utterance O No coding manual exists, description within (Friedlander, 1982)	C	B	C
Inventory of Therapeutic Strategies (ITS; Gaston and Ring, 1992)	Review of literature, review of therapy sessions (of cognitive, dynamic, and behavioral therapy) To assess the major intentions in therapist's interventions	19 items, 3 subscales: 1. explorative strategies (12 items, 3 content subcategories (defenses, emotions, cognitions), 4 objective subcategories (therapist, others, self, non-interpersonal situations) 2. supportive interventions (3 items), 3. work-enhancing strategies (4 items)	microanalytic: d, statement; global: m (5-point Likert scale), session O Coding manual exists as unpublished ms. (Gaston, 1988)	B	A	A
Helper Behavior Rating System (Shapiro et al., 1980), c.fr. (Barkham and Shapiro, 1986)	Elliott's (1979) Helper Behavior Rating System (Shapiro et al., 1984), review of existing rating systems To develop a system of response mode analysis for clinical research	12 categories: interpretation, exploration, reflection, general advisement, process advisement, reassurance, disagreement, open question, closed question, general information, self-disclosure, other	d, thought unit O Coding manual (Shapiro et al., 1980), available from the senior author	A	B	C
Response modes coding system (Connolly Gibbons et al., 2002)	Based on 6 published coding systems described within (Elliott et al., 1987) IPT, CT To compare and contrast the therapist response modes used in manual-guided interpersonal and cognitive therapy sessions	3 categories, 8 subcategories: 1. statement categories: learning statements, clarification, restatements, questions, informational/ directional statements, self-disclosures, role play, other statements 2. time frames: childhood, adult past, present, in session, future, unspecified 3. person code: father, mother, parents, sibling, immediate family, extended family, therapist, other real person, unspecified, significant other, child, patient	d, speaking turn O Manual existing as unpublished manuscript, description of categories within (Connolly Gibbons et al., 2002)	C	B	C
System for assessing therapist communications (SATC; Brunink and Schroeder, 1979)	System Of Analysis (SOA; Fiedler, 1950, 1951; Strupp, 1957; Carkhuff, 1969) To compare psychoanalytically orientated, behavior, and Gestalt therapists	6 dimensions: type of therapeutic activity (8 categories: facilitating communications, exploratory operations, clarification, interpret-tive operations, structuring, direct guidance, activity not clearly relevant to task of therapy, and unclassifiable), temporal focus (present, past), interview content focus (4 categories: client, thera-pist-client relationship, therapist self-disclosures, content irrelevant to therapy, degree of initiative, communication (5 categories: relevant task-oriented communi-cations, accurate, nonadditive comm., additive comm., inaccu-rate or subtractive comm., comm. irrelevant to therapy, therapeutic climate (4 categories: minimally supportive or emotionally neutral, supportive or giving, nonsupport-tive or withholding)	d (m for “degree of initiative,” 4 levels), utterance O No coding manual published; description of dimensions within (Brunink and Schroeder, 1979)	C	B	B
Therapist Behavior Code-Revised (TBC-R; Bischoff and Tracey, 1995)	(TBC; Forgatch and Chamberlain, 1982) Focus on categorization of therapist's behavior into directive and nondirective units	8 categories: support (10 items), teach (5 items), structure (3 items), question and information seek (4 items), confront and challenge (8 items), interpret and reframe (6 items), talk (1 item), facilitate (1 item); additional subcodes for question and interpret: directive vs. non-directive	d, speaking turn O Coding manual existent as an unpublished manuscript	B	B	C
Verbal Response Mode (VRM; Stiles et al., 1989)	First author's previous work CBT, PI To classify speech acts which concern what people do when they say something, without respect to speech content or to interactants' affective states (version for client and for therapist available)	8 categories: disclosure (D), edification (E), question (Q), acknowledgment (K), advisement (general/process) (A), confirmation (C), interpretation (I), reflection (exploratory/simple) (R) Each category is rated separately regarding interpersonal intent and grammatical form	d, utterance O Coding manual (Stiles, 1992)	A	A	C

*m, metric; d, dichotomous; O, observer; CT, cognitive therapy; CBT, cognitive-behavioral therapy; PI, psychodynamic-interpersonal therapy; IPT, interpersonal therapy. Evaluation criteria: A, no limitations; B, some restrictions; C, severe deficits (see text of the manuscript for details)*.

### Scale development and research questions

All measures have been developed using a top down approach based on theoretical considerations, therapy manuals, literature research, clinical experience, and expert discussion as well as already existing scales. Three measures (APS, see Table 2; YACS, see Table 3; and PQS, see Table 4) included bottom-up analyses comprising analyses of therapist's utterances and audio- or videotaped therapy sessions among other strategies within the scale development process but none of the measures was developed exclusively in a bottom-up procedure (see Tables 1–5, column 3).

The measures have been developed to address various research questions. These can be classified into five groups: 1. Six measures aim to assess the adherence and competence of the therapist regarding specific therapeutic orientations (e.g., ACS-SEC, see Table 1; CTS-R, see Table 3; SPRS, see Table 4; YACS, see Table 3). 2. Among the pantheoretical measures, five measures aim to distinguish between two or more therapeutic orientations (e.g., CPPS, see Table 4; CSPRS, see Table 4; CSTF, see Table 5; SATC, see Table 5). 3. Two measures aim to investigate microprocesses in psychotherapy such as patient therapist interaction (e.g., PIRS, see Table 2; and CIP, see Table 3), or to analyze the relationship between therapeutic techniques and therapy outcome (four measures, i.e., APS, see Table 2; CAPS, see Table 1; MIT, see Table 2; PIC see Table 2). 4. Eleven measures try to assess all possible verbal techniques in general (e.g., ITS, see Table 5; PQS, see Table 4) or within a therapeutic orientation (e.g., Coding of therapist statement, see Table 2; CTACS, see Table 3). 5. Finally, six measures focus on very specific aspects of psychotherapy, e.g., techniques used within the psychodynamic therapy of patients with borderline personality disorder (TVII, see Table 1), or focusing theoretically important constructs such as “interpretations” (TIRS, see Table 2).

### Description of subscales, categories, and items

**Figure 3:**
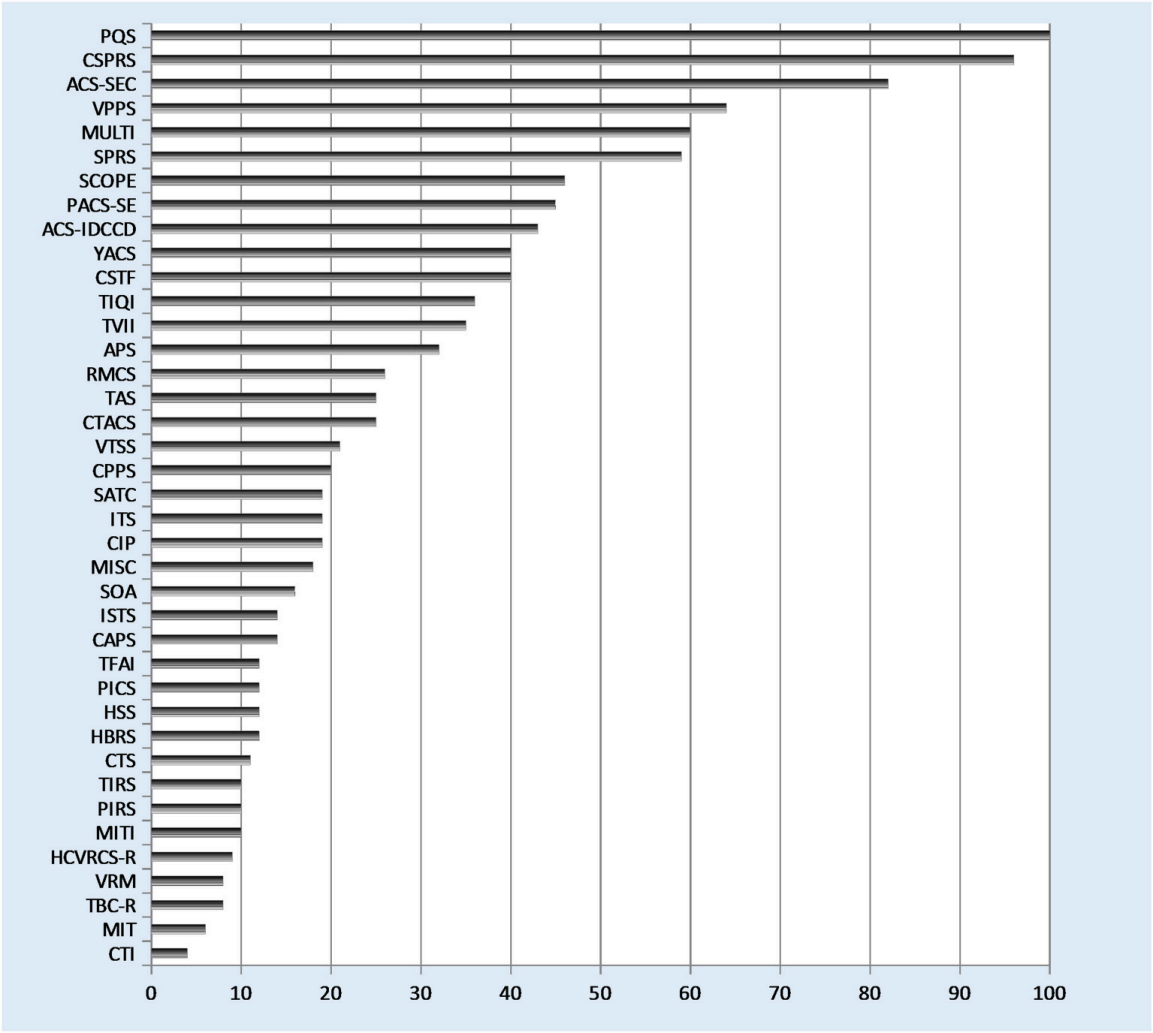
**Number of items per measure**.

According to the research questions, the measures assess verbal therapeutic techniques on various differentiation levels. The number of categories ranges from four categories without further differentiation (Coding of therapist statement; see Table 2; Connolly et al., 1998) to 100 items without further categorization (PQS; see Table 4; Jones, 1985) or 96 items, categorized into eight scales with several subscales (CSPRS; see Table 4; Hollon et al., unpublished work, cited from Hill et al., 1992; see Figure 3 for details). Apart from this differentiation level, the focus is partly limited to only one or two aspects of verbal techniques. The MIT (see Table 2) for example differentiates between “interpretation” vs. “non-interpretation.” The ISTS (see Table 1) and the ITS (see Table 5) distinguish the scales “supportive techniques” from “interpretative techniques” which include further items, respectively. The category or item that is assessed by the majority of measures is “interpretation.”

All measures assess techniques concerning their form (e.g., “question,” “interpretation,” “agenda setting”). Moreover, ten measures refer to the content of an intervention. Among those which address the thematic content or the temporal focus are for example the PIC (see Table 2; four content codes: defenses, anxieties, impulse/feeling, no content), the TAS (see Table 1; “future plans of patient”), and Response modes coding system (see Table 5; three categories: statement categories, time frames, and person code). But 12 measures also refer to the latent dimension, because they include scales or items which address the “climate or atmosphere” (PQS, see Table 4) or the “Therapist's warmth and friendliness” (VPPS, see Table 4). Some measures even mix the formal and latent dimension on item levels (e.g., VTSS, see Table 1: “Therapist shows evidence of listening respectively,” ISTS, see Table 1: “gratify the patient, i.e., make the patient feel good rather than anxious in the session”).

### Assessment mode and rating perspective

The assessment mode of the included measures can be generally classified into global vs. microanalytic. Global measures refer to whole therapy sessions or larger segments of sessions. Microanalytic measures refer to more fine-grained units like utterances or sentences. Furthermore, judged units can be assessed on metric vs. dichotomous scales and can be coded as manyfold vs. mutually exclusive.

Within the theoretical orientations, the amount of global and microanalytic measures is approximately counterbalanced. Global measures generally use metric scales, microanalytic measures prefer dichotomous items which are mutually exclusive. The only measures that assess verbal therapeutic techniques in a metric way on a microanalytic level are: 1. the APS (where ratings refer to segments of session so that it is not purely microanalytic, see Table 2) and one scale (“degree of initiative”) of the SATC (see Table 5), which does not belong to the observable formal or content dimension of verbal techniques. All in all, no measure purely measures verbal techniques in a metric way on a microanalytic level.

The PQS (see Table 4) is the one measure that uses a completely different rating method (Q-sort; Jones, 1985). The 100 items have to be sorted for each session on a continuum from 1 = least characteristic to 9 = most characteristic so that the distribution of items yield a normal distribution.

In general, all measures are based upon an external observer's rating; the only exception is the TIQI (see Table 4) which is rated by the therapist himself. Two measures (CPPS and MULTI, see Table 4) can be additionally rated by the therapist and the patient. These two measures therefore allow simultaneous ratings from three different perspectives.

### Psychometric aspects

#### Objectivity

Of the 34 included measures, only 14 report a detailed coding manual which is published and/or available in the World Wide Web or from the authors and therefore fulfill the A-criterion within this category. Within these coding manuals, the definitions of the categories or items are described in detail and rater instructions are given. Only two manuals are less exact, one gives general information about the structure and use of the measure (APS, see Table 2) and one manual only gives short definitions of the categories (PIRS, see Table 2). For eight measures, the coding manual exists as an unpublished manuscript. For 13 measures, no coding instruction is reported, but a description of the scales or categories can mostly be found in the published articles.

#### Reliability

There are large differences regarding the reliability of the included measures (see Tables 1–5, column 9 and 10; see Supplementary Material for procedure and results of reliability calculation). First, the procedures of reliability calculation differ widely. The number of sessions and patients on which the values are based ranges from three sessions with three patients (total of 9 sessions; Silberschatz et al., 1986) to eight sessions with 33 clients (total of 264 sessions; Stiles et al., 1989). Some studies report reliability scores based on consensus ratings (e.g., Butler et al., 1995) or ratings achieved by regular meetings to prevent rater drift (e.g., Gaston and Ring, 1992; Connolly Gibbons et al., 2002). Some reliability values are based on ratings of students, others on ratings of the measures' authors. In most cases, raters have been trained extensively before using the measure (e.g., Goldfried et al., 1997), but sometimes not (e.g., McCarthy and Barber, 2009). Some studies describe the rating procedure incompletely so that information about the number of rated sessions is missing.

Secondly, the reliability gold standard by calculating ICC's is fulfilled by *n* = 16 of the included measures. Within these, ICC (2,2) and ICC (2,1) are mostly used. For two measures, it remains unclear which form of ICC was calculated (VTSS; see Table 1, Butler et al., 1995; CTS-R; see Table 3, Blackburn et al., 2001). Overall, ICC values range from acceptable to high levels. Scales, categories or items with ICC values below the acceptable level are found in measures with discrete categories, so none of the measures had to be excluded because of lacking reliability, although ICC values are presented.

All other measures (*n* = 18) report Finn's r correlation coefficients, Pearson product-moment correlation coefficients or kappa values. Similar to ICC values, it is often not clearly specified which kind of kappa has been applied (e.g., “Light's kappa,” Light, 1971; “Fleiss kappa,” Fleiss, 1971), whether it is the median or the average kappa). One measure failed to report reliability values for all subscales. Since available values were good, the measure was not excluded from the review.

#### Validity

Six of the included measures report no information about the validity of the measure and three further measures report insufficient information. For three measures, studies are available which report a broad examination and evidence for validity for the respective measure: CPPS (see Table 4; Hilsenroth et al., 2005), ITS (see Table 5; Gaston and Ring, 1992), and YACS (see Table 3; Carroll et al., 2000). All other measures report validity on a minimal level, especially with regard to criterion validity (which means that scales reflect and distinguish between different therapeutic orientations) and convergent validity (which means that the scale has been compared with another measure).

All in all, only two measures fulfill the A-criterion for all three categories (CPPS, see Table 4, and YACS, see Table 3; see Supplementary Material for procedure and results of validity calculation).

## Discussion

The objective of the present study was to review available measures designed to assess directly observable (i.e., formal, temporal, and thematic) dimensions of verbal therapeutic techniques in a reliable and valid manner. All in all, 34 measures were identified which show a great heterogeneity regarding purposes, theoretical foundations and assessment procedures. Whereas there is much more consensus related to outcome measures in psychotherapy (e.g., Strupp et al., 1997). In general, it appears that most research groups rather created their own new measure than using existing ones in order to address their research questions. Explanations of this approach include that available measures are too specific in focus (McCarthy and Barber, 2009), do not cover the necessary constructs under investigation (Milbrath et al., 1999), require too much effort, or show insufficient psychometric properties (Hilsenroth et al., 2005). This practice, however, generated a number of problems in the assessment of verbal techniques.

### Issues regarding the systematization of techniques

The reviewed measures refer to a specific theoretical orientation or to different theoretical orientations. They have been developed to address different research questions such as assessing adherence and competence of the therapist regarding specific therapeutic orientations, distinguishing between therapeutic orientations, investigating microprocesses in psychotherapy, or analyzing the relationship between techniques and therapy outcome.

Some of them assess all possible verbal techniques (again within a therapeutic orientation or in general), whereas others focus on selected theoretically important constructs such as “interpretation.” “Interpretation” is the category or item that is assessed by the majority of measures. The focus is partly limited to only one or two aspects of verbal techniques (e.g., differentiation between “interpretation” vs. “non-interpretation” or “supportive techniques” vs. “interpretative techniques”). There are heterogeneous levels on which techniques are differentiated. The number of categories ranges from four categories without further differentiation to 100 items without further categorization.

A potential problem is that the hierarchical structures of the measures are different, although the same or similar techniques are assessed. For example, the ISTS (see Table 1) contains the scale “interpretative techniques” (level 1) which includes further subordinated items like the “impression of the therapist” (level 2). The HSS (see Table 5), on the other hand, subsumes items as “interpretation” (level 2) within the scale “insight skills” (level 1). Thus, it is difficult to specify the relationship of scales and items in both measures (notably the intervention “interpretation”) as well as results derived from using them.

The definitions of verbal techniques are also very different across measures. In some cases, the same categories are defined in different ways. For example, the category “interpretation” was defined as a therapeutic utterance which goes beyond the perceptions of the patient (Hill, 1978), an utterance which refers to one or several dynamic components (e.g., wishes, fears, defense mechanisms; Piper et al., 1998) or a theory-derived response of the analyst, rated according to the degree to which it transforms meaning by bringing aspects outside of awareness into full awareness (Waldron et al., 2004b). In other cases, intervention categories show overlaps and can hardly be distinguished from one another. This situation is certainly due to and aggravated by the fact that—in the theoretical literature—specific intervention techniques are described and defined inconsistently, abstractly and in part ambiguously, which limits their operationalizability and investigation in evidence-based therapy research (Fonagy, 2000; Paniagua, 2003; Gumz et al., 2014).

Another problem derives from the different facets of the assessment of verbal techniques. One problem in this respect is that many items or subscales of available measures simultaneously assess different features of therapeutic techniques (e.g., formal and qualitative features, thematic content, and therapist's intentions), prohibiting the possibility to analyze these features separately (e.g., VTSS, see Table 1: “Therapist encourages the patient's expression and/or exploration of feelings in relation to a significant other (including therapist.”)

From our point of view it is helpful to clearly specify on which level techniques are to be described. There is a difference between settings in which more global descriptions are needed (e.g., role play technique, sculpture work, hypnosis or transference regarding a more comprehensive topic) and settings in which a focus on the microanalytic level of single verbal interventions is more important (e.g., the verbalization of emotional reactions or a specific transference interpretation; Gumz et al., 2014) and where there is greater overlap between different therapeutic schools. Furthermore, it would be beneficial to separate the basic features of verbal techniques (Form, Temporal focus, Thematic content, Latent characteristics). This relates specifically to the latent characteristics. This would provide the opportunity to evaluate different aspects of therapeutic techniques, i.e., to differentiate between what the therapist said and what his utterance implied. Even if this strict systematic may be difficult to realize in research it may be useful to compare results and conduct meta-analyses.

### Issues regarding assessment mode and rating perspective

Half of the measures reviewed (*n* = 17) use global assessment (i.e., rating of complete sessions) while the other 17 measures use a microanalytic assessment referring to different units like utterances, sentences or speaking turns. The advantage of global assessment is that they allow for individual weighting of information (Bøgwald et al., 1999) and that they are time-saving, thereby allowing the analysis of larger sets of data. On the other hand, global methods deliver rather crude assessments of complex processes and are, moreover, prone to cognitive biases, e.g., anchoring effects and availability heuristics (Tversky and Kahneman, 1974; see also Heaton et al., 2010). Microanalytic assessments, on the other hand, are more labor-intensive which limits the amount of data to be analyzed. However, microanalytic methods allow for the analysis of the effects of specific intervention techniques on a micro-level which is to date hardly known and would help to clarify the processes involved in psychotherapeutic change. In summary, both assessment modes have advantages as well as disadvantages that need to be considered before conducting a study. A general desideratum in this respect will be to define what “a verbal intervention” actually is, i.e., which unit (single sentence vs. speaking turn vs. therapy session) needs to be assessed.

Another issue refers to the rating perspective. Except for three measures (TIQI, CPPS, MULTI, see Table 4), all included measures have to be used by an external observer. This perspective may be sufficient to answer many research questions regarding verbal techniques. Some topics, however, cannot be covered by analyzing external ratings, e.g., differences in the perception of verbal techniques by therapist vs. patient. Accordingly, it would be helpful to conceive measures which allow the assessment of different perspectives on the therapy process to increase the range of analytic possibilities.

### Issues regarding psychometric aspects

A final set of problems relates to the psychometric properties of the measures reviewed. Objectivity is the least problematic aspect in this respect: For most measures (*n* = 22) the authors reported the availability of explicit coding manuals. Some of these manuals were published (*n* = 14) which makes it possible to reconstruct the rating process in detail (A-criterion). In unpublished manuals (*n* = 7), it is more difficult to draw firm conclusions about the objectivity of the rating process. However, it can be assumed that the ratings within published studies are based on manuscripts describing the use of the measures so that objectivity is given on a minimal level (B-criterion). For the remaining measures (*n* = 13), no manuals were reported so that the objectivity of the rating process is not guaranteed.

The reliability of the measures reviewed is much more difficult to evaluate, since different authors used a wide range of different methods of reliability assessment. For *n* = 16 measures ICC values were reported and classified as sufficiently reliable (A-criterion). The remaining measures (*n* = 18) show a satisfying reliability reporting kappa, Fleiss' *r* or product-moment correlations. It was observed that reliability values in some cases should be treated with caution, because the rating procedures were described incompletely, the exact method of reliability assessment was not specified (e.g., “kappa” instead of “Cohen's kappa” or “Fleiss” “kappa”), or the occurrence of interrater discussions which may lead to a falsifying increase of reliability over time. The evaluation of the validity of results proved to be the most problematic aspect in the assessment of verbal techniques. Only three measures (CPPS, see Table 4; ITS, see Table 5; YACS, see Table 3) revealed good values for different fields of validity while for all other measures there is only minimal or hardly any information related to validity. The question remains, why the important validation criterion is not considered sufficiently within the scale development process.

All in all, only two measures fulfilled the A-criterion in matters of objectivity, reliability and validity, i.e., the CPPS (see Table 4, which allows to discriminate between two or more therapeutic orientations) and the YACS (see Table 3, which aims to assess the adherence and competence of the therapist in treatments for substance use disorders). From a methodological perspective, these are the measures which can be recommended for clinical use regarding the respective research questions without reservation. All other measures show more or less severe psychometric deficits regarding psychometric aspects. From a practical perspective, some of these measures might nevertheless be helpful to address specific research questions, particularly to analyze the relationship between therapeutic techniques and therapy outcome or to investigate microprocesses in psychotherapy. However, in general it needs to be stated that further research has to operate with more refined methods ensuring the objective, reliable and valid rating of verbal therapeutic techniques.

### Limitations

It could be possible that the used keywords did not identify all instruments that assess verbal techniques. Another limitation is that only papers in English and German language were considered which excludes measures in other languages. Investigating the effect of techniques on therapeutic outcome was outside the scope of this review, and would be a valuable contribution for future research.

### Are the proposed standards reasonable or a pie in the sky?

Our systematic review revealed a number of problems regarding the measures available for the assessment of verbal techniques. Due to these problems of assessing verbal techniques, it is almost impossible to compare the scales and categories of different measures as well as results derived from studies, even if they examine the same outcome variable. Accordingly, no definite conclusions regarding their efficacy can be drawn.

Where does this leave us? In this review, we have certainly used very high standards for measures of psychotherapy research. It must be discussed if these standards are really reasonable or if they are “pie in the sky,” since they are difficult to accomplish and may be too rigorous. Certainly, these issues might be painfully obvious to many investigators. On the other hand, it is important and useful to reiterate these criteria to enhance progress in research and to reach standards common in psychotherapy outcome research (e.g., Strupp et al., 1997). Some of the authors of the studies we rated as deficient, undoubtedly might have provided interesting and reasonable justifications as to why they did not attain what we refer to as the “gold” standard. Moreover, we have also listed some problems which cannot be avoided or need to be tolerated. Nevertheless, a solid theoretical as well as a methodological framework for measuring verbal techniques using clear terminology and accurate conception is of paramount importance to advance this field of research.

Language is the cornerstone of most psychotherapy sessions. Knowing which specific verbal techniques “good” therapists use in their practical work has a high practical relevance for psychotherapist training and clinical routine. Many questions remain in the domain of studying verbal techniques. We need more sophisticated theoretical knowledge regarding the question which techniques really matter and which should be classified as “clinical lore” (Barber, 2015, p. 325). Moreover it is important to know what the specific features of a particular technique actually are. And it will be important to achieve a consensus concerning the definition and operationalization of verbal techniques. Regarding these topics more discussions between clinicians and researchers are necessary. An optimal solution would be to define techniques without reference to therapeutic methods and to find clear unambiguous designations for specific techniques so that the same thing is not called by different names. Authors of measures need to clarify the specific features of verbal techniques that are to be assessed. Although the four basic features which we suggested (*Form, Temporal focus, Thematic content, Latent characteristics*) are not always easily distinguished, it may be worth the effort, as this would finally help to disentangle the complex mechanisms involved in the efficacy of verbal techniques.

What is the importance of “specific” techniques which are considered to belong to a particular psychotherapy method compared to other techniques which are used without reference to a particular method? In order to answer this question the creation and use of multitheoretical scales is of great theoretical and practical utility (Barber, 2015, p. 232). The question can be broadened: To what extent is the outcome due to unintended or even non-theoretically relevant techniques (Barber, 2015). Answering this latter question it would be useful to describe what therapists do without any reference to a specific theory. For this purpose, bottom-up, qualitative approaches could be helpful to develop scales in order to be able to gather theoretically unbiased, non-predetermined and comprehensive information.

Another interesting research question would be how the latent features of verbal techniques complement and interact with the directly observable features in explaining good outcome, or how the use of different therapeutic techniques changes during the course of therapy.

A significant proportion of the outcome variance is explained by a therapist effect (Wampold, 2001). Our understanding of the variables responsible for these effects of the therapist appears to be lacking (Castonguay, 2011). It may be particularly interesting to study the possible interaction between personal characteristics and therapeutic techniques. Apart from focusing on who the therapist is, what he/she should do in therapy to facilitate change is likely to be of great interest to clinicians (Castonguay, 2011).

A final important question would be how the techniques interact with alliance or other common factors to bring about patient improvement. Castonguay (2011) named the investigation of the interaction between participants, relationship, and technique variables for different clinical disorders as one of the two most important directions of future research.

Answering these clinically relevant questions can be facilitated by establishing a theoretically clear system and accurate conceptions as well as a methodically solid basis.

## Conclusions

To date, the results regarding verbal interventions gathered with available instruments can hardly be compared. This has mainly three reasons: 1. Different objectives for the use of the instruments (e.g., measuring competence and adherence vs. differentiation between therapy methods vs. analyzing specific theoretical constructs), 2. Insufficient systematization (e.g., analysis of different or different numbers of interventions, partly on different hierarchical levels), and 3. Inconsistencies in the definition of categories (definitions with the same label often do not match, or vice versa, identical aspects have differing designations).

In conclusion, the overview over the status quo of assessment methods allows for the formulation of important desiderata of future research. Firstly, it will be important to achieve a consensus concerning the definition and operationalization of verbal techniques.

Secondly, we suggest that authors of measures need to clarify the specific features of verbal techniques that are to be assessed. In our review, we applied a typology comprising four features, i.e., the *Form*, the *Temporal focus*, the *Thematic content*, and *Latent* characteristics. Although these four features are not always easily distinguished, it may be worth the effort, as this would result in a more focused approach toward verbal techniques which would finally help to disentangle the complex mechanisms involved in the efficacy of verbal techniques. It would reduce the chaos and complexities of definitions and make it easier to find an orientation among concepts and empirical findings.

Thirdly, it will be important to put more emphasis on the psychometric properties of measures. Our review revealed that only two of the available measures fulfill the highest standard of the three central validation criteria. In general, authors of future measures need to provide explicit coding manuals ensuring the objectivity of the rating process and demonstrate the reliability and validity of the assessment. Finally, our review revealed that to date there is no measure which was primarily developed in the course of a qualitative bottom-up approach, i.e., starting from empirical data of therapeutic utterances in therapy sessions which are then explicitly described and classified. For future research, we recommend that this approach should be granted more attention, as it allows the gathering of comprehensive information (Schreier, 2012).

Given that future research meets these basic desiderata, it should be possible to establish a firmer ground for the assessment of verbal therapeutic techniques. This would, as a consequence, advance the state of knowledge about the question which techniques can help whom with specific sets of symptoms in certain situations, and finally help to specify empirically based guidelines for psychotherapeutic practice.

## Author contributions

AG conceived and coordinated the study. BT and HW participated in the design of the study. All authors (AG, BT, BS, CM, HW) substantially contributed to the acquisition, analysis, or interpretation of data for the work. AG, HW, and CM drafted the manuscript. All authors revised it critically for important intellectual content. All authors gave their final approval of the version to be published. All authors gave their agreement to be accountable for all aspects of the work in ensuring that questions related to the accuracy or integrity of any part of the work are appropriately investigated and resolved.

## Funding

This research received no specific grant from any funding agency in the public, commercial, or not-for-profit sectors.

### Conflict of interest statement

The authors declare that the research was conducted in the absence of any commercial or financial relationships that could be construed as a potential conflict of interest.
