# Supplementary material for: Measuring Verbal Psychotherapeutic Techniques—A Systematic Review of Intervention Characteristics and Measures
Source: Front Psychol. 2015 Nov 10;6:1705. doi: 10.3389/fpsyg.2015.01705 (PMC4639607; doi:10.3389/fpsyg.2015.01705)
Supplement: Supplementary file 1 [file DataSheet1.DOC]

**Supplementary material**

**Procedure and results of reliability and validity calculation**

Global measures referring to psychodynamic therapy

| **instrument**  **source** | **procedure of**  **reliability and**  **validity calculation** | | **results of reliability and validity** | |  |
| --- | --- | --- | --- | --- | --- |
|  |
| **ACS-SEC**  **Adherence/Competence Scale for SE for Cocaine Dependence**  developed within the NIDA-CCTS  Barber et al., 1997  Barber et al., 2004 | 2 doctoral-level clinical psychologists (experts in SE-therapy) rated 52 audiotaped sessions of 49 patients (32 SE, 10 CT, and 10 IDC therapy sessions), raters received a great deal of training  raters should be experts of the treatment modality | | ICC (2,2) for AD/COM =  .74/.62 (supportive)  .89/.49 (expressive)  .89/.25 (cocaine abuse)  .85/.43 (total scale score)  internal consistency (Cronbach’s alpha): >.65 for all subscales  criterion validity could be demonstrated for the adherence scale: scale distinguishes between different therapeutic modalities | |  |
| **CAPS**  **Columbia Analytic Process Scale**  Vaughan et al., 1997 | 2 raters (2 of the authors) rated 16 transcript sessions of 9 patients | | kappa = .5 (SD = .21)  construct validity couldn’t be demonstrated by two factor random effects linear model, because of a lack of consensus about the definition of AP (biggest amount of the variance was due to ‘error term’) | |  |
| **ISTS**  **Interpretive and Supportive Technique Scale**  Ogrodnizcuk & Piper, 1999 | 2 raters (bachelor’s-degree-level, randomly chosen of 10 raters) rated one session of 50 patients (total of 50 sessions) after training (didactic overview of psychodynamic theory, group discussion of coding manual and scale, several practice sessions) | | ICC (2,1) on item level between .25 (impression of others) and .90 (interpretations)  Scales:  .93 (supportive techniques), .88 (interpretative techniques), .95 (full scale)  internal consistency (Cronbach’s alpha):  >.65 for both scales  criterion validity: scales reflect therapeutic orientation; convergent validity could be demonstrated by comparison with TIRS | |  |
| **TAS**  **Therapist Action Scale**  parallel form: PAS - Patient Action Scale  Hoyt et al., 1981 | 5 psychodynamically trained judges rated (after 12 hrs. of training) each 40 audiotaped therapy sessions of 25 patients (total of 100 sessions) | | Finn’s r (median) = .76 (Range = .44 - .92)  construct validity could be demonstrated by a cluster analysis for the two scales TAS and PAS | |  |
| **TVII**  **Therapist Verbal Interventions Inventory**  Königsberg et al., 1985 | 3 raters rated after 5 hrs. of training 7 videotaped 15-minute-segments of therapy sessions of 1 patient | | Finn’s r = .42 - .99  no information about validity | |  |
| **VTSS**  **Vanderbuilt Therapeutic Strategies Scale**  developed within the Vanderbilt Psychotherapy Research Project  Butler, Henry, & Strupp, 1995 | | 4 advanced clinical psychology graduate students rated 15-minute-segments in pairs (consensus rating) of 22 patients | | ICC = .44 - .71 (IS); .76 - .91 (SS)  internal consistency (Cronbach’s alpha):  >.65 for both subscales  convergent validity could be demonstrated by comparison (Pearson product moment correlations) with relevant subscales of the VPPS | |

Notes. m: metric; d: dichotomous; O: observer; AD: adherence, COM: competence; SE: supportive-expressive dynamic psychotherapy, AP: analytic psychotherapy, NIDA-CCTS: National Institute on Drug Abuse - Collaborative Cocaine Treatment Study, VPPS: Vanderbilt Psychotherapy Process Scale, ICC: intraclass Coefficient correlations, CT: cognitive therapy, IDC: individual drug counseling

Microanalytic measures referring to psychodynamic therapy

| **instrument**  **source** | **procedure of**  **reliability and**  **validity calculation** | **results of reliability and validity** |
| --- | --- | --- |
| **APS**  **Ananlytic process scales**  Waldron, et al., 2004 | *Coding manual (former version):*  3 raters rated 2 sessions with 31 patient segments and 25 therapist segments | “alpha correlational statistics” = .75 (encourage elaboration), .52 (clarify), .77 (interpretation), .49 (provide support), .70 (patient’s defense), .71 (patient’s reaction to the analyst/analytic situation), .82 (patient’s conflicts), .72 (developmental focus), .37 (self-esteem issues), .66 (confronting), .79 (feelings of the analyst), .63 (follow the patient’s immediate emotional focus), .67 (good intervention)  face validity: clinical experience, suggestions of clinicians; convergent validity explored by comparison with Q-set-items: “alpha” correlational statistics show correlations of .37 - .89 between Q-set- and APS-items |
| **CTS**  **Coding of therapist statement**  Conolly et al., 1998 | 3 therapists (familiar with CTS) rated transcripts of 3 sessions of 33 patients (total of 98 sessions)  Connolly et al., 1999:  3 sessions of 29 patients were rated by 3 independent raters (total of 86 sessions; unknown, if audio- or video-taped or transcripts) | ICC (2,3) =  .54 (interpretation)  .79 (clarification)  .90 (questions)  .95 (other)  ICC (2,3) = .66 - .83  convergent validity could be demonstrated by comparison with PACS-SE |
| **MIT**  **Malan intervention typology**  Malan, 1963, cited f. Silberschatz et al., 1986 | 4 clinical judges rated verbatim transcripts of 3 sessions of 3 patients (total of 9 sessions) | kappa = .76/.77  no information about validity |
| **PIC Psychotherapy Interaction Coding System**  McCullough, 1988, cited f. Town, 2012 | 8 doctoral level clinical psychology trainees or graduates rated after 16 hrs. of training 10-minute video-taped segments of 6 patients (24 segments each, total of 144 segments) | mean Cohen’s kappa for individual ratings = .56  no information about validity |
| **PIRS Psychodynamic Interventions Rating Scale**  Cooper & Bond, 1992, cited from Milbrath 1999 | 2 raters (2 of the authors) rated transcripts of therapy sessions of 20 patients (total of 20 sessions)  Hersoug, Bogwald, & Hoglend, 2005: 2 trained raters rated transcripts of the 7^th^ and 16^th^ therapy session of 39 patients (78 sessions) | Light´s kappa = .85 (87% agreement; Range = .83 - .99 across the categories)  ICC (2,2) for the 7^th^/16^th^ session =  .78/.79 (interpretive interventions)  .97/.98 (supportive interventions)  construct validity demonstrated by a sequential analysis of therapist interventions and patient elaboration  Milbrath et al., 1999 |
| **TIRS**  **therapist intervention rating system**  Piper, Debbane, de Carufel, & Bienvenu, 1987 | 2 raters (bachelor’s-level psychology majors) scored each 1 audiotaped session of 15 patients (total of 15 sessions) | mean kappa k = .71 (range = .42 - .94)  average Pearson product-moment-correlation r = .76 (range = .51 - .96)  no information about validity |

Notes. PACS-SE: Penn Adherence-Competence Scale for Supportive-Expressive therapy, ICC: Intraclass Coefficient Correlations

Measures referring to cognitive-behavioral orientated therapy or to a specific setting

| **instrument**  **source** | **procedure of**  **reliability and**  **validity calculation** | **results of reliability and validity** |
| --- | --- | --- |
| **CTACS**  **Cognitive Therapy Adherence-Competence Scale**  developed within the NIDA - CCTS  Liese et al., 1995, cited from Barber, Liese, & Abrams, 2003 | cognitive therapists with 5 years of experience rated 134 audiotapes therapy sessions of 129 patients (total of 134 sessions) (monthly telephone conferences to prevent rater drift)  raters should be experts of the treatment modality | ICC (2,2) = .37 - .93 (AD)  ICC (2,2) = .22 - .94 (COM)  internal consistency (Cronbach’s alpha) =  >.65 for both scales  criterion validity could be demonstrated by separate contrast analyses comparing CT to SE and IDC conditions |
| **CTS-R**  **Cognitive Therapy Scale**  Young & Beck, 1980, cited from Blackburn et al., 2001, diverse revisions, among others by Blackburn et al., 2001 | Blackburn et al., 2001:  3 raters rated 3 videotaped sessions of 34 patients (total of 102 sessions) | ICCs for pairs of raters = .40 - .86  internal consistency (Cronbach’s alpha):  >.65 for both scales  face validity could be demonstrated by expert ratings; criterion validity could be demonstrated by an increase of competence over time during a training |
| **CIP**  **Coding system for the Interaction in Psychotherapy**  Schindler, Hohenberger-Sieber & Hahlweg, 1989 | 10 transcribed intake sessions of 64 persons independently coded by 3 postgraduate psychology students and 3 expert raters; intensive 50-hour training; periodic training sessions to prevent rater drift; reliability is based on comparison of each rater with expert rating | Cohen’s kappa =  .80 (therapists; SD = 0.06)  .79 (patients; SD = 0.11)  content validity has been tried to be shown by comparison of the beginning and the ending part of standardized interviews; no further information about validity |
| **ACS-IDCCD**  **Adherence/ competence scale for IDC for cocaine dependence**  developed within the NIDA - CCTS  Barber, et al., 1996 | 4 ICD experts rated 41 audiotaped IDC-sessions of 40 patients  raters should be experts of the treatment modality | ICC (2,2) for AD/COM = .82/.77 (monitoring drug use behavior), .73/.62 (encouraging abstinence), .83/.72 (encouraging 12-step participation), .83/.55 (relapse prevention), .70/.67 (educating the client), .84/.75 (total score)  internal consistency (Cronbach’s alpha):  >.65 for all subscales  criterion validity demonstrated by separate contrast analyses: scales reflect therapeutic orientation (IDC, SE, CT) |
| **SCOPE**  **Sequential Code for Observing Process Change**  Moyers & Miller, 2006 | Hannöver et al., 2013:  3 coders rated randomly each 2 of 32 transcript counseling sessions; raters were trained about 40 hrs. | Cohen’s kappa for each pair of coders for all categories: k = .590 - .822  predictive validity demonstrated by effects of specific coded categories on an in-session level |
| **YACS**  **Yale Adherence and Competence Scale II**  Carroll et al., 2000 | 5 raters rated each 19 tapes (total of 95 sessions), randomly selected of 741 sessions of 122 patients;  training until raters reached good rater reliability, rater recalibration sessions are held regularly to prevent rater drift; raters should be experienced clinicians | ICC (2,1) for AD/COM = .80/.85 (assessment), .83/.71 (general support)  .85/.83 (goals), .95/.98 (CM), .93/.93 (TSF), .88/.88 (CBT)  criterion validity: scales reflect therapeutic orientation; concurrent validity could be demonstrated by correlation of general support subscales with alliance; factor structure could be demonstrated by confirmatory factor analyses (AD subscales) |

Notes. CT: cognitive therapy, PI: psychodynamic-interpersonal therapy, IPT: interpersonal therapy, CM: clinical management; ET: exploratory therapy, ICC: Intraclass Coefficient Correlations, AD: adherence, COM: competence, CBT: cognitive behavioral therapy, CM: clinical management, TSF: twelve step facilitation, IDC: individual drug counseling, SD: standard deviation, SE: supportive expressive therapy, NIDA-CCTS: National Institute on Drug Abuse - Collaborative Cocaine Treatment Study.

Pantheoretical global measures

| **instrument**  **source** | **procedure of**  **reliability and**  **validity calculation** | **results of reliability and validity** |
| --- | --- | --- |
| **CPPS**  **Comparative Psychotherapy Process Scale** Hilsenroth, 2005 | 2 raters (advanced clinical psychology graduate students) rated 80 videotaped sessions; raters had supervised training (15 sessions); regular reliability meetings were held during the coding process to prevent rater drift | ICC (2,1) = .93 (PI subscale), .82 (PI mean, range: .66 - .91), .95 (CBT subscale), .82 (CBT mean, range: .67 - .95)  ICC (2,2) = .97 (PI subscale), .89 (PI mean, range: .85 - .95), .98 (CBT subscale), .90 (CBT mean, range: .79 - .97)  convergent validity demonstrated by correlation (Pearson r) with VTSS and PACS-SE; criterion validity demonstrated by analyses of variance: scales reflect therapeutic orientation; clinical utility demonstrated |
| **CSPRS**  **Collaborative Study Psychotherapy Rating Scale (Form 6)**  Hollon et al., 1984, cited from Hill et al., 1992 | rotating teams of two raters (advanced doctoral students) rated an equivalent number of tapes of 28 therapists  Hill et al., 1992: principal component analysis based on four sessions of 180 patients | ICC (2,8) = .88 (CBT), .78 (IPT), .80 (CM), .47 (FC)  .58 (ED)  internal consistency (Cronbach’s alpha) = >.65 for all scales  the findings of Hollon et al. (1984) couldn’t be replicated; criterion validity: scales reflect therapeutic orientation |
| **SPRS**  **Sheffield Psycho-therapy Rating Scale**  Shapiro & Startup, 1990, cited from Startup & Shapiro, 1993 | 220 audiotaped therapy sessions are rated by 2 of 8 raters each (graduate psychologists) after 50 h of training, periodic meetings to prevent rater drift | ICC (1,1) = .78 (ET), .85 (P), ICC (1,2) = .65 (FC)  internal consistency (Cronbach’s alpha) = >.65 for all scales  criterion validity: scales reflect therapeutic orientation; convergent validity demonstrated by correlation with Therapist Session Intention (TSI)  (Stiles et al., 1996) |
| **MULTI Multitheoretical List of Therapeutic Interventions**  McCarthy & Barber, 2009 | 60 untrained raters (57 students, 3 experts) rated 20 videotaped psychotherapy sessions directly after a lecture about the mechanisms of change in a particular theoretical orientation; raters were not blind to the orientation of the tape | ICC (A, 2) of experts/students = .83/.72 (BT), .89/.65 (CT), .64/.52 (DBT), .83/.59 (IPT), .70/.56 (PC), .79/.53 (PD), .78/.46 (PE), .68/.64 (CF)  internal consistency (Cronbach’s alpha):  >.65 for all scales  The a priori model of MULTI subscales fit the data reasonably but not parsimoniously in confirmatory factor analyses; criterion validity: scales reflect theoretical orientation |
| **PQS**  **psychotherapy process Q set**  Jones, 1985, cited from Jones, Krupnik, & Kerig, 1987 | 5 judges, transcripts of audio taped sessions of 40 patients (total 40 sessions), each session rated by 3 judges; raters were members of the research team, met periodi-cally to compare their ratings  Jones & Pulos, 1993: 10 judges (different theoretical orientation) rated 3 videotaped sessions of the same patient | Pearson product-moment correlation coefficient corrected with the Spearman-Brown formula r = .63 - .90 (median = .86)  internal consistency (Cronbach’s alpha):  >.65 for all items  (Ablon & Jones, 1999)  several Q-set items differentiate between different therapy orientations which shows construct and discriminant validity of the instrument |
| **TIQI-T / -P**  **Therapist Interven-tions and Qualities Inventory- therapist form / - patient form**  Bøgwald et al., 2001 | Pearson product-moment correlation between ratings of 23 therapists and those of 153 patients | mean r = .23 (range = -.09 - .88, SD = .19)  principal component analysis revealed 8 components which failed adequate internal consistency (Cronbach’s alpha: <.65 for 4 of 8 components)  validity (factor structure, distinction of theoretical orientation) couldn’t be demonstrated |
| **VPPS**  **Vanderbuilt Psychotherapy Process Scale**  O’Malley, Suh & Strupp, 1983; Strauß et al., 1992 | 2 raters (PhD psychologists) rated the first 3 audiotaped therapy sessions of 38 patients (4 were excluded, total of 100 sessions), judges compared their ratings on every 10th tape in order to prevent rater drift | Pearson product-moment correlation coefficients r =  .91 (client participation), .92 (client hostility), .86 (therapist warmth and friendliness), .79 (negative therapist attitude), .94 (client exploration), .94 (therapist exploration), .92 (client psychic distress)  internal consistency (Cronbach’s alpha): >.65 for all items  predictive validity demonstrated by multiple regression analyses regarding treatment outcome |

Notes. BT: behavioral therapy CT: cognitive therapy DBT: dialectical- behavioral therapy PC: person centered therapy PD : psychodynamic therapy, PE process experiential therapy, CF: common factors, FC: facilitative conditions, P: prescriptive therapy, ED: explicit directiveness, CBT: cognitive-behavioral therapy, PI: psychodynamic-interpersonal therapy, IPT: interpersonal therapy, CM: clinical management; ET: exploratory therapy, ICC: Intraclass coefficient correlations; VTSS: Vanderbilt Therapeutic Strategies Scale, PACS-SE: Penn Adherence-Competence Scale for Supportive-Expressive therapy.

Pantheoretical microanalytic measures

| **instrument**  **source** | **procedure of reliability and**  **validity calculation** | **results of reliability and validity** |
| --- | --- | --- |
|
| **CSTF**  **Coding System of Therapeutic Focus**  Goldfried, 1989, cited from Kerr, 1992 | Goldfried et al., 1997:  always 2 of 6 advanced graduate students coded 2 sessions of 57 clients treated by 5 therapists (raters had 60 - 90 hr of training; weekly or biweekly meetings to prevent rater drift) | ICC (1,1) = .59 - .95 (components), .64 - .88 (general interventions), .59 - .83 (intrapersonal), .62 - .78 (interpersonal), .60 - .98 (persons involved), .54 - .98 (time frame)  criterion validity: scales reflect therapeutic orientation; concurrent validity demonstrated by correlation with TFAI (Samoilov et al., 2000) |
| **HSS**  **Helping Skills System**  Hill & O’Brien, 1999, cited from Hess et al., 2006 | 3 judges (master’s degree students in counselor education) rated 4 videotaped vignettes after 2 training transcripts and discussion of judges after 8 ratings  Elliott et al., 1987:  phi-statistics about pairs of raters (co-authors and undergraduate students) of 7 therapy sessions | average kappa between pairs of judges = .91  Phi = .58 (approval/reassurance), .73 (closed question), .78 (open question), .59 (restatement)  .48 (reflection), .55 (confrontation/challenge), .62 (interpretation), .56 (self-disclosure), .94 (minimal encourager/immediacy), .73 (information), .61 (direct guidance)  criterion validity: scales reflect therapeutic orientation; convergent validity demonstrated by comparison with 5 other instruments |
| **HCVRCS-R Hill Counselor Verbal Response Cate-gory System - Revised**  Friedlander, 1982 | two raters rated parts of 34 counseling sessions of 17 clients and 11 doctoral student counselors | kappa = .83  minimally adequate face and content validity |
| **ITS**  **Inventory of Therapeutic Strategies**  Gaston & Ring, 1992 | 2 clinical judges (postdoctoral clinicians) rated 3 sessions of 16 patients, recalibration sessions to prevent rater drift | ICC (2,2) for cognitive/dynamic therapy = .85/.79 (exploratory strategies), .93/.66 (supportive interventions), .89/.64 (work-enhancing strategies); range overall: ICC (2,2) = .21 - .98  recalibration sessions caused stabilization and not improvement of reliability  criterion validity: scales reflect therapeutic orientation; content validity demonstrated by comparison with guidelines of therapy orientation; discriminant validity demonstrated by comparison with an instrument assessing the alliance |
| **Helper Behaviour Rating System** Shapiro, Barkham, & Irving's, 1980, cited from Barkham &  Shapiro, 1986 | Barkham & Shapiro, 1986:  3 coders (first author, 2 postgraduate research students) rated videotaped counselor sessions of 24 clients; regular meetings were held to prevent rater drift; raters had 20 hrs. of training | Fleiss‘ kappa (3-way) = .59 (interpretation), .53 (exploration), .70 (reflection), .72 (general advisement), .65 (process advisement), .95 (reassurance), .57 (disagreement), .72 (open question), .72 (closed question), .75 general information, .76 (self-disclosure), .53 (other)  no information about validity |
| **Response modes coding system** Conolly- Gibbons et al., 2002 | 7 graduate students rated after training each 3 of 548 sessions from 72 patients  (monthly recalibration session to prevent rater drift) | kappa = .72  ICC (2,3) = .71-.95 (statement categories), .70-.97 (time frames), .43-.97 (person code)  criterion validity couldn’t be demonstrated: instrument didn’t differentiate between IPT / CT |
| **SATC System for assessing therapist communications**  Brunink & Schroeder, 1979 | 3 PhD clinical psychologists rated verbatim transcripts of 18 sessions of 18 patients (total of 18 sessions) | intercorrelations (product-moment-correlation) between raters: r = .81/.82  criterion validity: scales reflect therapeutic orientation; convergent and discriminant validity could be demonstrated by comparison with ISTS-transference |
| **TBC-R Therapist Behavior Code - Revised**  Bischoff & Tracey, 1995 | 2 coders ((under-)graduate psychology students) rated after 16 hrs. of training 10 videotapes of 4 patients | kappa = .57  to prove content and face validity, experts assessed the scale in regard to different aspects; no further information about validity |
| **VRM**  **Verbal Response Mode**  Stiles & Shapiro, 1989 | 5 coders rated 8 audiotaped sessions from 33 clients (total of 264 sessions)  40-60 hrs. of training (manual, training transcripts, discussion of ratings), weekly meetings to prevent rater drift  reliability based on 84 session, rated by 2 independent raters | ICC (1,1) for form/intent = .97/.69 (D), .92/.87 (E), .98/.90 (Q)  .93/.94 (K), .83/.96 (A), .88/.42 (C), .96/.84 (I), .68/.73 (R)  no information about validity |

Notes. m: metric; d: dichotomous; O: observer; CT: cognitive therapy, IPT: interpersonal therapy, ICC: Intraclass coefficient correlations, TFAI: Therapeutic focus on Action and Inside; ISTS: Interpretive and Supportive Technique Scale, D: disclosure, E: edification, , Q: question, K: acknowledgment, A: advisement, C: confirmation, I: interpretation, R: reflection.
